# Factors Associated with Mental Health among Malaysian University Music Students: Roles of Fear of COVID-19, Nomophobia, Loneliness, Sleep Quality, and Socioeconomic Status

**DOI:** 10.3390/healthcare11010018

**Published:** 2022-12-21

**Authors:** Chunmei Zhuang, Hashem Salarzadeh Jenatabadi

**Affiliations:** 1 Faculty of Creative Arts, University of Malaya (UM), Kuala Lumpur 50603, Malaysia; 2 Department of Science and Technology Studies, Faculty of Science, Universiti Malaya, Kuala Lumpur 50603, Malaysia

**Keywords:** music students’ mental health, moderation analysis, quality of life, sleep quality, depression

## Abstract

Previous mental health studies have shown higher levels of anxiety, stress, and depression symptoms among university music students. In general, some similar findings have been observed for Malaysian music university students. In diagnosing the complications of mental health, there is consensus that it is essential to develop and evaluate a model oriented toward mental health illness prevention and treatment. To date, a suitable pattern for estimating mental health in terms of anxiety, stress, and depression among music university students is lacking. To fill this gap, we collected the necessary data from 691 music and 871 general students who were students for one year. The introduced pattern includes socioeconomic status, fear of COVID-19, nomophobia, sleep quality, loneliness, and mental health. Our data analysis proved that the levels of anxiety, depression, and stress of music students were lower than those of general students. Unlike some previous studies, in this study, the fear of COVID-19 and nomophobia didn’t have the most significant impact on mental health. The most significant impacts were related to sleep quality and loneliness. These findings have the potential to inform health promotion and services in the music education system.

## 1. Introduction

There is strong evidence that university students in a variety of fields of study are vulnerable to mental illness [1,2]. College students experience a higher incidence of physical and psychological sickness than the general population, according to a study by Henning and Krägeloh [3]. Moreover, it has been discovered that university students suffer one or two mental diseases, such as anxiety and depression [4]. According to a different study by Suarez, Cardozo [5], anxiety symptoms are reported by 44.9% of medical students and 6.8% of the general population, while depression symptoms are recorded by 33.9% and 2.6% of people. Existing research suggests that music students have higher rates of stress, anxiety, and depression than their non-musical counterparts. Spahn, Strukely [6] compared four groups: 266 medical students and 247 music students, as well as 71 psychology and 71 athletics majors. The authors found that music students face higher levels of anxiety and depression compared to the other students. Araújo, Wasley [7] compared the health-promoting behaviors and general health of music (*n* = 208) and general (*n* = 65) students in the United Kingdom and found that the former group had lower levels of self-regulation and self-efficacy.

According to the findings of the studies cited above, even before the widespread spread of the COVID-19 virus, there have been growing worries in recent years regarding the mental health of college students. Students in higher education institutions are more likely to suffer from mental health issues as a direct result of the stressors and constraints brought on by the pandemic. This may have a detrimental effect on their academic performance, social relationships, and future opportunities in the professional and personal spheres. After a year and a half of the pandemic, this suffering among university students might be significant [8].

Previous research has taken a fragmented approach to investigating the elements that affect mental health. These studies have focused on certain aspects of mental health, such as social media [9], physical activities [10], technostress [11], nomophobia and sleep quality [12], anxiety and sleep quality during the pandemic [13,14], and socioeconomic background [15]. This study, on the other hand, takes a more holistic approach by taking into account a number of different aspects that are still significant, such as fear of COVID-19, nomophobia, loneliness, sleep quality, and socioeconomic status. The majority of the earlier studies that looked at these important components were carried out in a variety of research settings, with a general range of student populations and a narrow focus on particular aspects of the situation. As a result, the current study added to the body of literature by investigating the factors using a framework. Additionally, we took mental health into account as a latent variable, which is a combination of stress, anxiety, and depression. The study also takes place in a previously unstudied Malaysian musical and general educational context. Additionally, this investigation used Bayesian Structural Equation Modelling. It is employed to model the causal link between variables and identify the model’s most significant influencing factors. The results of such a comprehensive approach will make it easier to create the most effective strategies for addressing many stakeholders, including educators, higher education institutions, and the government, in order to further promote mental health. With consideration of the above objectives, the present study aims to answer the following questions:

What is the status of anxiety, depression, and stress among Malaysian music university students and general students?

What are the rules of fear of COVID-19, nomophobia, sleep quality, loneliness, and SES in estimating mental health among Malaysian music and general university students?

## 2. Literature Review and Theoretical Framework

Fear has been the most prevalent psychological reaction in the populace throughout the current COVID-19 outbreak [16]. People are concerned about their health, so this response or reaction is crucial [17]. It is the most frequent physiological and mental response to significant societal or social problems, such pandemics or emerge, which results in psychologically uncomfortable behaviours [18]. It is also linked to high levels of anxiety, which can cause irrational thought. Such circumstances can lead to mental health problems in the general public and in universities during a pandemic scenario. Consequently, concern over COVID-19 may be a sign of mental health (including anxiety, stress, and depression). Additionally, it was discovered that nomophobia, or the fear of not being able to access and enjoy information through mobile phones, is one of the modern world’s diseases that is already evident in young people [19].

Nomophobia, which is the fear of not having a phone or phone contact, was previously recognised as a modern disorder [20]. It is a problem that Yildirim and Correia [19] characterised as having three dimensions: (i) anxiety over losing contact with others, (ii) inability to get information through phones, and (iii) lack of convenience of smartphone applications. The dependence on cell phones has significantly expanded during the COVID-19 epidemic [21] as they are a crucial communication tool and offer additional advantages such as access to online courses, playing video games, listening to music, etc. If people are unable to use their smartphones, all of these activities could make people feel much more anxious [22]. According to a previous study by Tams and Legoux [23], nomophobia will cause stress if withdrawal control from the phone is poor or event uncertainty is high. In addition, Farooqui and Pore [24] have demonstrated that people are more likely to suffer fear, panic, depression, and anxiety when they are unable to access their smartphone. Additionally, empirical research conducted by Samaha and Hawi [25] provided support for the concept by demonstrating that people who suffer from nomophobia experience tension whenever their smartphones are not within reach. Given that the fear of COVID-19 and nomophobia have comparable symptoms, the effect that fear of COVID-19 has on mental health may be made worse by nomophobia. Those students who spent a significant amount of their time engaging in compulsive internet use, gaming addiction, and excessive use of social media reported high levels of despair, loneliness, poor sleep quality, and anxiety [26].

One of the world’s most widespread adult difficulties is loneliness. Those who suffer from it have a “persistent and unfavourable psychological condition characterised by a sense of emotional isolation, a sense of being alone, and a sense of estrangement from others” [27]. The negative emotion known as loneliness results from a mismatch between the number of friends one has and the number of friends one would want to have [28]. Fan, Chen [29] presented that feelings of isolation, estrangement, and dissatisfaction in interpersonal connections characterise loneliness. These negative emotions have been linked to an increase in mortality and other health hazards [30]. Loneliness, for instance, is associated with clinical disorders such as stroke and cardiovascular sickness and is a predictor of psychological symptoms like despair, stress, and worry [31].

A good night’s sleep is one of the most important factors in determining how effectively one can carry out the activities of daily living and how well one can function mentally [32]. During the epidemic, students had to make adjustments to their lifestyles and confront several problems, including adjusting to a modified teaching-learning structure, dealing with the loss of social connectedness, and more. This could have a detrimental impact on the quality of sleep that they get [33], and it could also lead to elevated levels of depression, anxiety, and stress [34]. Because good quality sleep is necessary for effective neurocognitive and psychomotor functioning as well as for optimal physical and mental health, poor sleep quality may lead to difficulties with attention and poor academic performance [35]. There is a correlation between poor sleep quality and a number of parameters, including psychological factors, physical activity, lifestyle factors, and chronic disorders [36].

## 3. Materials and Methods

### 3.1. Search Method

The online database from the Web of Science was used to search the literature on the mental health of music university students for works published between 2000 and 2022. Both “mental health” and “music students” were used as key phrases. A built model using the Bayesian SEM method was presented. The primary terms we searched from the start were “music students”, “depression”, “anxiety”, “stress” and “structural equation modelling”. We were aiming for “Fear of COVID-19” with “nomophobia” in the second phase. We combined “student mental health” with “loneliness”, “socioeconomic” and “sleep quality” for the third phase.

### 3.2. Study Design

A quantitative cross-sectional methodology was used for this investigation. From January 2022 through July 2022, the survey was fielded. The Malaysian government issued a nationwide movement control order that effectively ended on-campus, face-to-face instruction at all levels of education from kindergarten through university. Most Malaysian college students had already taken three online courses, either in real-time or asynchronously.

### 3.3. Sampling

In this article, we used a cross-sectional analysis in which the required sample size is relevant to data collection at one point in time. According to Hair et al. [37], the sample size needed in the research should be related to the number of latent variables of the study which include the number of indicators inside the latent variables as described below:

- A minimum of 100 respondents with five or fewer latent variables, each of which must have three indications, are required.
- A minimum of 150 respondents with seven or fewer latent variables, each of which must have three indications, are required.
- 300 respondents are required, with certain latent variables having no more than three indications and no more than seven latent variables.
- 500 respondents are needed, and there are more than seven latent variables, some of which have fewer than three indications.

Our research framework includes six latent variables, and the educational level is categorized into two groups. Therefore, we expected to have at least 100 respondents for every group (music and general students). In total, we needed 200 respondents.

There was a concerted effort made to ensure sample consistency throughout this investigation. Ethical approval from the University of Malaya Research Ethics Committee (UMREC) was obtained prior to beginning data collection (UM.TNC2/UMREC 1582). Informed consent was obtained from all respondents after an explanation of the study’s goals was provided. Respondents were emailed a Google form survey with several pre-set questions. Some of them were given out by their university course instructor. A total of 1562 responses were received from the survey. All protocols and laws were strictly adhered to while conducting the research.

### 3.4. Instrument

Questionnaires were distributed among the participants to achieve the research goals. The information on the participants was organized into a few categories based on the variables which include personal SES, Fear of COVID-19, nomophobia, loneliness, sleep quality and mental health. To describe the SES variable, we used three indicators: age group, working experience, and income per month. The age group of the participants is classified into five criteria, which are ‘less than 21 years old’, ‘between 21 to 25 years old’, ‘between 26 to 30’, ‘between 31 to 35 years’ and ‘more than 35 years old’ which coded to 1, 2, 3, 4 and 5, respectively. Respondents are asked about their duration of working experience where the responses are coded as 1 for ‘no job experienced’, 2 for ‘between 1 to 3 years’, 3 for ‘between 4 to 6 years’, 4 for ‘between 7 to 10 years’ and 5 for ‘more than 10 years’. The responses to income per month are coded as 1, 2, 3, 4 and 5 for ‘less than RM 1000’, ‘RM 1000 to RM 2000’, ‘RM 2000 to RM 3000’, ‘RM 3000 to RM 4000 and ‘over RM 4000’, respectively [RM: Ringgit Malaysia]. Subjects’ mental health was evaluated using the DASS-21 [38], which has been found to be reliable and valid. There are 21 items in total; 7 each for stress, anxiety, and depression. A total DASS-21 score can be anywhere from 0 to 63, with a subscale score ranging from 0 to 21 and an item score ranging from zero (did not apply to me at all) to three (applied to me very much). Respondents’ sleep quality was evaluated using the Pittsburgh Sleep Quality Index (PSQI) [32], a self-administered questionnaire that evaluates sleep quality over the preceding month. There are 19 parts which are arranged into seven components: subjective sleep quality, sleep latency, sleep duration, sleep efficiency, sleep disturbance, use of sleep medication, and daytime dysfunction. Loneliness was measured using a modified version of the Likert scale of Russell, Peplau [39], which has four points, ranging from ‘never’ to ‘always’. Nomophobia was evaluated using an adapted version of the 7-point Likert scale that was developed by Yildirim and Correia [19]. The scale ranges from 1 (Definitely do not agree) to 7 (Definitely agree).

### 3.5. Statistical Method

From a mathematical and statistical modelling point of view, regression (bivariate or multivariate) [40,41] and ANOVA [42,43] are the most familiar techniques used for analyzing the associations between various factors and mental health among music university students. Structural Equation Modelling (SEM) has garnered a lot of attention in the last few decades thanks to studies that show its potential in the field of mental health [44]. This method makes it possible to estimate psychological well-being as a result of causal relationships (simple or complex) between observable and non-observable (latent) factors. Previous research has introduced several estimators for use in SEM analysis. Maximum likelihood (ML) is the estimator of choice for studies involving SEM analysis [45]. However, model misspecification is a common problem that undermines ML applications. Examples of overly restrictive models include those that require no residual correlations and exact zero cross-loadings [46]. It has been shown by Kolenikov [47] and Asparouhov and Muthén [48] that the ML estimator has a large parameter bias in factor correlations and factor loadings. Researchers have begun using alternative estimators in modelling to get around the shortcomings of ML in SEM analysis brought on by factors such as small sample sizes and the normal distribution of independent variables. Very few researchers have proposed replacing the ML estimator with a Bayesian one in SEM analysis [49], but this may be the key to getting around ML’s limitations.

We used the SEM technique in this research because it has helped researchers to understand the concept of latent variables and the interactions within the model. SEM has the following features which are an advantage when applying this technique for mental health modeling.

- The ability to use latent variables, a feature of SEM that is unique in that they cannot be observed directly and are not used by other analysis techniques [50].
- The capacity to calculate and analyse the direct and indirect links between the research study’s variables [51].
- The capacity to demonstrate relationships between dependent variables suggests the estimation of multiple exogenous and endogenous variables simultaneously [52].

### 3.6. Research Framework

**Figure 1:**
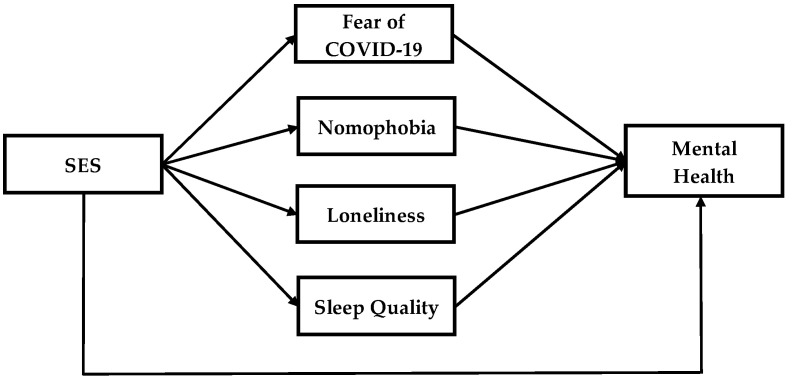
Research framework.

The framework of the study is presented in Figure 1. The research framework of this study is being created by the combination of the theoretical framework with the addition of some new ideas. Independent and dependent variables in this research relate to each other by using the mediator to gain the output. The research framework includes six latent variables. Socio Economic Status (SES) is presented as an independent variable and mental health as a dependent variable. Our framework contains four mediators which are fear of COVID-19, nomophobia, loneliness and sleep quality. This research is delivered through a cross-sectional study design.

## 4. Results

### 4.1. Descriptive Statistics

At 15 universities in Malaysia, an online poll was conducted. Table 1 provides descriptive statistics for mental health status as well as the number of questionnaires that were completed at each educational level. We measured the severity of stress, anxiety, and depressive symptoms among Malaysian music and general university students using descriptive statistics.

### 4.2. SEM Analysis

#### 4.2.1. Validity and Reliability

According to Fornell and Larcker [53], to test the validity and reliability of a survey, some conditions for SEM analysis need to fit. To examine the validity, every latent variable in the research should be equal to or higher than 0.7 for the Cronbach’s alpha value. From Table 2, the Cronbach’s alpha value for every latent variable is aligned with the condition that reinforces the validity of this research.

Next, to test the reliability of this research, every latent variable indicator should have a factor loading value higher than 0.7.

Hence, after the elimination of indicators from research data, claiming the reliability of the research must comply with another condition i.e., all latent variables should have an equal or higher than 0.5 value of the average variance extracted (AVE). Table 3 shows the AVE output for this research, which indicates that the AVE of every latent variable was higher than 0.5. As the features of reliability are fulfilled, this research is confirmed.

#### 4.2.2. Model Fitting

The output of SEM model fitting is tabulated in Table 4. The range in the model fitting analysis that is endorsed should be above 0.9 to determine the suitability of the research model. The comparative fit index (CFI), normed fit index (NFI), relative fit index (RFI), incremental fit index (IFI), goodness of fit index (GFI), and Tucker Lewis index (TLI) value of the chosen BMI group levels were within acceptable ranges. Therefore, the model fitting of three groups of data is accepted.

#### 4.2.3. Structural Model

**Figure 2:**
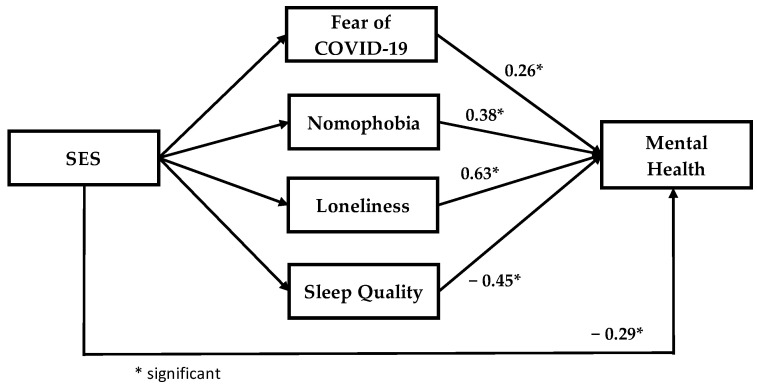
Output of mental health model (music students).

**Figure 3:**
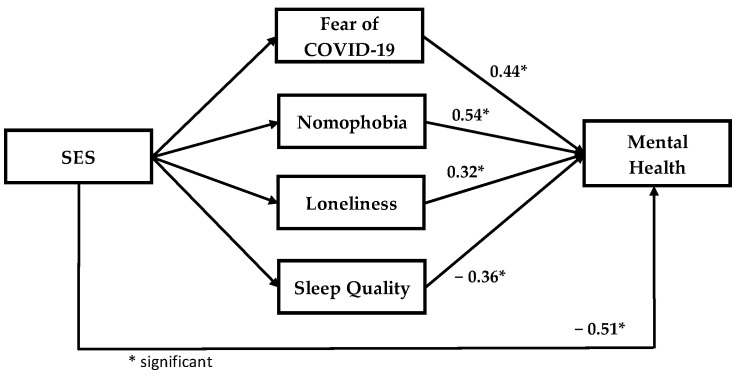
Output of mental health model (general students).

In the SEM analysis, we used the structural model to recognize the substantial connection between research variables that are linked to the considered conceptual model. Figure 2 and Figure 3 show the output of the structural model for the music and general students. Based on Figure 2 and Figure 3, the impact of each latent variable on mental health are significant for music and the general students’ model.

## 5. Discussion

During the course of their education, students go through a substantial transition unlike any other, from high school to university or college [54]. This transition marks the beginning of a significant period of psychological and social development, which can have a variety of effects on students’ lives, including their health [55]. However, in the past two years, COVID-19 has caused massive disruptions throughout the world. The majority of university learning and evaluation processes have shifted from face-to-face courses to distance and/or online learning, which has made it more difficult for students to access learning and adjust to the new techniques. Second, students are separated from the general public. During a pandemic, they must remain at home, even if they are unable to see their families for many months. Thirdly, the students had financial difficulties. Due to the lockdown, many undergraduate and graduate students who previously worked to earn extra money are now unable to do so. All of these concerns may have contributed to an increase in stress, affecting anxiety and depression. Young university students may experience symptoms of depression as a result of the stress caused by these long-lasting, major changes, or they may first experience a condition of worry that could later progress to mental health issues.

The purpose of this study was to investigate the factors that influence the mental health (anxiety, depression, and stress) of music and general college students, specifically focusing on fear of COVID-19, nomophobia, sleep quality, loneliness, and socio-economic connections.

The findings indicated that of the variables shown to have a significant causal association with students’ mental health, among music students, sleep quality status had the most direct negative relationship, with greater sleep quality scores being related with lower mental health (DASS scores). Silva et al.’s discovery of an independent link between mental health and sleep quality status among medical [56] and arts [57] students is supported by these findings. Poor sleep quality is associated with increased tension, irritability, anger, depression, anxiety, and confusion, according to a study conducted on university students during COVID-19 post epidemic era by Tang, Chen [58]. Therefore, university students with sleep quality issues still face multiple severe sources of stress, increasing their risk for developing or worsening existing mental health issues.

In our research, there is a strong positive correlation between loneliness and mental health. The outcomes concurred with earlier research showing that loneliness had strong significant relationships with mental health [59], including depression [60] and anxiety [61]. These results provide credence to Kim and Cho [62] that people with insecure attachments struggle to fully control their emotions and may experience feelings of loneliness because they lack healthy attachments. This result highlights the need to concentrate on various approaches to minimize social isolation and raise knowledge of the advantages of social relationships. In the specific situation of COVID-19, some of the possibilities could include making regulations on community interactions more flexible if the health prevention changes can be implemented, or promoting the creation of “safe social bubbles” in which a group of individuals with more controlled external interactions can gather with a limited number of people as long as everybody respects the health-preventive recommendations [63]. These options have the potential to reduce the risk of the spread of COVID-19 [63].

According to the results of our research, 78.4 percent of music students suffer from some degree of nomophobia. There were 16.3% of people with mild phobia, 53.12% with moderate, and 8.98% with severe phobia. These levels are lower than those found in previous investigations among teachers [51] and high school students [64]. Furthermore, the current study found that the third most important factor that significantly impacted mental health was nomophobia. Several studies demonstrate that nomophobia has a substantial impact on the mental health of university students. Farchakh, Hallit [28] demonstrated a substantial association between nomophobia and anxiety, depression, stress, and impulsivity. Moreover, Tung, Gan [65] revealed that students with severe nomophobia had greater anxiety and distress levels. A possible explanation is that a person with high levels may utilize a smartphone to research coping strategies for dealing with COVID-19. For example, if a student has greater levels of nomophobia in the context of COVID-19, as suggested by fear of COVID-19, the student may be driven to keep, protect, and construct a variety of resources in order to survive and sustain well-being. On the other side, stress and anxiety enhance the likelihood of phone addiction. Before COVID-19, a prior study by ÖZDEMİR, ÇAKIR [66] reported students with higher Internet addiction scores were more worried than those with lower scores. Those who reported high levels of gaming addiction, compulsive internet use, and social media use also reported significant levels of depression, avoidance, poor sleep quality, loneliness, and depression-related anxiety [26].

SES is the fourth important characteristic that has a detrimental effect on the mental health of music students. Lower socioeconomic status was related to greater DASS scores. This conclusion is consistent with the findings of Fernandes, Biswas [26], who found an independent association between mental health and socioeconomic status among Medical Sciences students. However, according to their research, SES has the most detrimental effect on mental health. However, according to our research, socioeconomic status has the fourth greatest impact on the mental health of musicians. A lower socioeconomic position is associated with a greater burden in a variety of aspects of day-to-day life as well as an increased exposure to stressful life situations. Therefore, unpleasant life events and other stressors are obviously related to socioeconomic position, and it is clear that lower parental education and lower family income were associated with higher stress levels regardless of whether or not the individuals in question were university students.

Fear of COVID-19 is the subject of numerous studies about the mental health of university students. COVID-19 had the least significant impact on mental health status among the five predictors examined in this study. In this study, fear of COVID-19, among music students, had a lesser effect on mental health than four other predictor variables: SES, sleep quality, loneliness, and nomophobia. However, this does not imply reduced worry of COVID-19. Young university students’ fear of COVID-19 may have been strongly influenced by the quick spread of the disease, the enormous number of individuals affected, the rising number of fatalities, mistrust of the health care system, ignorance, and disinformation. This fear has been identified as a factor that affects depression, and the effects of this fear on depression may be exacerbated by the presence of anxiety. The majority of students live away from their families, are unable to return, and/or reside in poor conditions where it is difficult to remain healthy and make ends meet, causing them to experience more fear when they perceive they are in a more precarious and dangerous situation.

## 6. Conclusions

According to the findings of this study, music students’ perceptions of uncertainty and risk might serve as a fertile breeding ground for stress, anxiety, and depression, resulting in a mental health concern. Recognizing the complexity of the interactions between these elements and developing mental health symptoms, we must rapidly develop intervention programs in universities to assist these music and general students in better coping with this type of situation. We found that the levels of anxiety, depression, and stress of music students are lower than those of general students. This study developed a framework for music students’ mental health, which improved based on previous studies related to SES, fear of COVID-19, loneliness, nomophobia, and sleep quality. The most significant impacts among music students belonged to loneliness and sleep quality. However, among general students, the most significant impacts belong to nomophobia and SES. Nevertheless, a few limitations to this research have occurred as follows:

- Some studies involved “family chronic illness” and “mental wellbeing history” for analyzing anxiety, depression, and stress among university students [67,68]. However, we didn’t consider it in this research, but we think that this is one of the indicators that affects the mental health of the respondents.
- The study’s validity may be jeopardized because the data was self-reported. Because this method has been widely used in previous studies, we are confident that the data obtained are of high quality, and our data collectors stressed the confidentiality of all answers.
- Nomophobia may cause someone who is afraid of COVID-19 to use a smartphone to look for online help regarding their mental health. This should be researched further.
- We used nomophobia in our research model. “Technostress” is a well-known indicator that has been used in previous studies related to university students’ academic performance [11], sleep quality [69], anxiety and depression [70]. It could be significant to use it for estimating university students’ mental health.
- It would be prudent to repeat the study during later phases of the pandemic and under normal conditions, as our study may have been a snapshot geared toward coping with COVID-19 in Malaysia’s last phases.
- This article describes a new study that used Bayesian SEM to examine the mental health of music university students at various educational levels. In the SEM technique, the significance of the variables is very important in measuring the strength of the relationship between variables. This would be a beneficial addition to future research to expand knowledge of mental health among music university students, which is becoming a serious issue that needs to be addressed.

## Figures and Tables

**Table 1:** Descriptive statistics of mental health.

Criteria		Stress		Anxiety		Depression	
Music students (number Percentage) Total = 691							
4.21–5.00	Very High	109	15.8%	106	15.3%	120	17.4%
3.41–4.20	High	164	23.7%	128	18.5%	136	19.7%
2.61–3.40	Medium	166	24.0%	176	25.5%	165	23.9%
1.81–2.60	Low	165	23.9%	201	29.1%	187	27.1%
1.00–1.80	Very Low	87	12.6%	80	11.6%	83	12.0%
General students (number Percentage) Total = 221							
4.21–5.00	Very High	161	18.5%	169	19.4%	184	21.1%
3.41–4.20	High	271	31.1%	196	22.5%	284	32.6%
2.61–3.40	Medium	283	32.5%	284	32.6%	251	28.8%
1.81–2.60	Low	113	13.0%	186	21.3%	61	7.0%
1.00–1.80	Very Low	43	4.9%	37	4.2%	91	10.5%

**Table 2:** Cronbach’s alpha output.

Research Variables	Music Students	General Students
SES	0.77	0.77
Fear COVID-19	0.73	0.71
Nomophobia	0.79	0.74
Loneliness	0.81	0.80
Sleep Quality	0.76	0.75
Mental Health	0.78	0.71

**Table 3:** AVE output.

Research Variables	Music Students	General Students
SES	0.55	0.63
Fear COVID-19	0.58	0.65
Nomophobia	0.59	0.52
Loneliness	0.61	0.58
Sleep Quality	0.64	0.65
Mental Health	0.62	0.66

**Table 4:** Model fitting analysis.

	Music Students	General Students
CFI	0.913	0.921
NFI	0.909	0.937
RFI	0.945	0.944
GFI	0.911	0.912
IFI	0.977	0.904
TLI	0.932	0.909

## Data Availability

The datasets generated and analysed during the current study are available from the corresponding author.
